# A field study evaluating the activity of N8‐GP in spiked plasma samples at clinical haemostasis laboratories

**DOI:** 10.1111/hae.13813

**Published:** 2019-07-11

**Authors:** Stefan Tiefenbacher, Wan Hui Ong Clausen, Martin Hansen, Rasmus Lützhøft, Mirella Ezban

**Affiliations:** ^1^ Laboratory Corporation of America Holdings Colorado Coagulation Englewood Colorado USA; ^2^ Novo Nordisk A/S Søborg Denmark; ^3^ Novo Nordisk A/S Måløv Denmark

**Keywords:** chromogenic assay, clinical laboratory techniques, factor VIII activity assay, haemophilia A, one‐stage assay, postadministration monitoring

## Abstract

**Aim:**

N8‐GP (turoctocog alfa pegol) is a glycoPEGylated, extended half‐life human recombinant factor VIII (FVIII) shown to be an efficacious treatment for patients with haemophilia A. Accurate monitoring of replacement therapy helps ensure proper dosing, leading to better patient care. The objective of this field study was to evaluate the accuracy and intra‐ and inter‐laboratory variabilities of N8‐GP and rAHF (Advate^®^) FVIII activity (FVIII:C) measurements in clinical laboratories using their routine methods and reagents.

**Methods:**

Laboratories measured plasma samples spiked with 0.03, 0.2, 0.6 and 0.9 IU/mL N8‐GP or rAHF. Samples were blinded, and laboratories were instructed to perform evaluations using their routine FVIII activity assays and calibrators.

**Results:**

Of the 67 participating laboratories from 25 countries, 60 used a one‐stage assay, 36 used a chromogenic assay, and 29 used both one‐stage and chromogenic assays. Participating laboratories used nine different activated partial thromboplastin time (aPTT) reagents, the most common being SynthASil^®^ and Actin^®^ FS. Most aPTT reagents recovered N8‐GP close to target. Three silica‐based aPTT reagents (APTT‐SP, TriniCLOT™ and STA^®^ PTT‐Automate) underestimated N8‐GP, recovering 40%‐83% of target concentration. For chromogenic assays, N8‐GP and rAHF recoveries were comparable at all concentrations, with overall mean recoveries for both products close to 130%. Assay variability was similar for both assay types and both products; inter‐laboratory variability was greater than intra‐laboratory variability and highest at 0.03 IU/mL.

**Conclusions:**

Most clinical laboratories accurately measured N8‐GP and rAHF when using their in‐house one‐stage or chromogenic FVIII:C assays. However, three silica‐based aPTT reagents underestimated N8‐GP recovery.

## 1 INTRODUCTION

N8‐GP (turoctocog alfa pegol; Novo Nordisk A/S) is a glycoPEGylated extended half‐life (EHL) recombinant factor VIII (rFVIII) molecule under investigation for the prevention and treatment of bleeding episodes and surgical management of patients with haemophilia A (HA). Attachment of a 40‐kDa polyethylene glycol (PEG) moiety to an O‐glycan in the truncated B‐domain of turoctocog alfa (NovoEight^®^; Novo Nordisk A/S) extends the half‐life of the molecule by 1.6‐fold in adults 1 and 1.9‐fold in children.^2^ Upon activation of N8‐GP, thrombin cleaves the FVIII B‐domain, with the attached PEG moiety, which is released from the remaining molecule, leaving the primary native structure of activated FVIII intact.^3^ Results from clinical studies show that N8‐GP is efficacious in the treatment of patients with HA and shows a favourable safety profile in children,^2^ adolescents and adults.^1^, ^4^

Accurate monitoring of FVIII activity (FVIII:C) during replacement therapy helps determine the dosing regimen and maintain trough levels in the target range. To monitor FVIII:C levels in patients with HA, clinical laboratories currently use activated partial thromboplastin time (aPTT)‐based one‐stage clotting assays, chromogenic activity assays or both. The most common method used to monitor patients treated with replacement therapy in clinical laboratories is the one‐stage clotting assay.^5^ One‐stage assays estimate FVIII:C during the clotting phase of the reaction using aPTT reagents that vary in the contact activators used to initiate clot formation.^6^ In vitro evidence suggests that some aPTT reagents can influence FVIII:C measurement of EHL‐FVIII products.^7^ A two‐centre study found that most aPTT reagents reliably recover N8‐GP in spiked plasma samples. However, of the eight reagents evaluated in the study, one aPTT reagent (APTT‐SP [Instrumentation Laboratory]) underestimated FVIII:C and was judged unsuitable for patient monitoring.^8^

Another method used to measure FVIII:C in the clinical laboratory is the chromogenic assay. Although the European Pharmacopoeia (Ph. Eur.) recommends that manufacturers of FVIII products assign potency using a chromogenic assay, this assay is less commonly used in clinical laboratories.^9^, ^10^, ^11^ N8‐GP potency was assigned using the Coamatic^®^ (Chromogenix; Instrumentation Laboratory) FVIII chromogenic assay and an in‐house reference material traceable to the World Health Organization (WHO) 8th international FVIII concentrate standard (IS) (National Institute of Biological Standards and Control [NIBSC]).^1^, ^12^ The potency assignment for N8‐GP has since been verified using six different chromogenic kits from alternative vendors with no significant difference in recovery of N8‐GP.^12^

Accurate FVIII:C monitoring is necessary to ensure optimal patient care. Thus, it has been recommended that assays used to measure FVIII:C in patients treated with EHL‐FVIII products should be validated at the individual laboratory prior to use.^13^, ^14^ In general, the objective of a haemophilia replacement product field study is to assess the methods and suitability of reagents currently in use for measuring FVIII:C in new products in order to give guidance to the clinical laboratories on the assay performance of specific reagents.^9^, ^11^, ^15^, ^16^, ^17^ This global comparative field study evaluates the accuracy and intra‐ and inter‐laboratory variabilities of FVIII:C measurements in clinical laboratories when using their routine FVIII:C procedures for measurement of N8‐GP and the unmodified rFVIII molecule, rAHF (Advate^®^, Shire Plc). Field study kits of congenital haemophilia A plasma samples spiked with a range of concentrations of N8‐GP and rAHF were distributed to participating clinical laboratories. Participating laboratories responded to a survey about reagents and methods they routinely use to monitor FVIII:C and measured the field study kits using their routine one‐stage clotting assay, chromogenic assays or both.

## 2 MATERIALS AND METHODS

Invitation letters for this study were sent to laboratories that participated in a previous field study 9 and to laboratories affiliated with the External quality Control for diagnostic Assays and Tests (ECAT) Foundation. Participating laboratories completed an online questionnaire, indicating their routine methods, kits and reagents used to measure FVIII:C, and were sent a field study kit.

### 2.1 Field study samples

Field study kits were prepared by Esoterix Inc and contained samples consisting of pooled congenital HA donor plasma (Pool‐3651; George King Bio‐Medical Inc) spiked with 0.03 (very low), 0.2 (low), 0.6 (medium) or 0.9 IU/mL (high) N8‐GP (Lot ER40146) or rAHF (Advate^®^; Lot E‐15‐05032). As a control, a vial of the International Society on Thrombosis and Haemostasis (ISTH) Scientific and Standardization Committee (SSC) secondary coagulation standard lot #4 plasma (National Institute of Biological Standards and Control [NIBSC] code: SSCLOT4) with the assigned FVIII:C value of 0.88 IU/vial was included in each study kit. Samples were frozen immediately after dilution at ≤−70°C and sent to laboratories by temperature‐logged transport. Laboratories were blinded to the product and exact concentration. However, samples were marked to indicate the expected FVIII:C as ‘very low’, ‘low’, ‘medium’ or ‘high’. Three colour‐coded replicates of each vial were provided, and laboratories were instructed to measure each sample on a separate day according to colour code. Esoterix Inc and the Laboratorium für Klinische Forschung GmbH verified factor activity in spiked sample sets prior to initiation of the study. Laboratories were instructed to perform FVIII:C analyses using their routine FVIII:C procedures, reagents, calibrator and instruments.

### 2.2 Statistical analysis

Each laboratory analysed the samples based on local practice. When more than one analysis using the same assay and methodology was performed by a clinical laboratory, the average was calculated. FVIII:C measurements are reported as IU/mL or per cent of target concentration based on actual potency. FVIII:C levels were log‐transformed and analysed separately by assay using a mixed effect model with the combination of trial drug and concentration as fixed effect and laboratory/assay as a random effect. The mean estimates of each concentration level together with the 95% confidence intervals (95% CI) were back‐transformed and presented alongside the inter‐ and intra‐laboratory variation. The same model was also used to analyse the concentration as percentage of target, based on the calculated percentages without any transformation. All data presented in histogram and scatter plots were prepared by per cent target concentration. The acceptable range of recovery was considered ±30% of the expected target concentration. All statistical analyses were performed using sas ^®^ 9.4, with sas/stat ^®^ 13.2 software.

## 3 RESULTS

### 3.1 Participating laboratories

In total, 67 laboratories from 25 different countries participated, including laboratories from France (16.4%), USA (11.9%), UK (10.4%), The Netherlands (7.5%), Australia (6.0%), Canada (6.0%) and Japan (6.0%). Sixty laboratories (89.6%) used FVIII one‐stage clotting assays, 36 laboratories (53.7%) used FVIII chromogenic assays, and 29 laboratories (43.3%) used both one‐stage and chromogenic assays. Full details of the geographic distribution of laboratories by assay type are summarized in Table S1. Of the laboratories that used both one‐stage and chromogenic assays, 17 (58.6%) used the same calibrator for both assays, while 11 (37.9%) used different calibrators.

### 3.2 One‐stage assays and aPTT reagents

**Figure 1:**
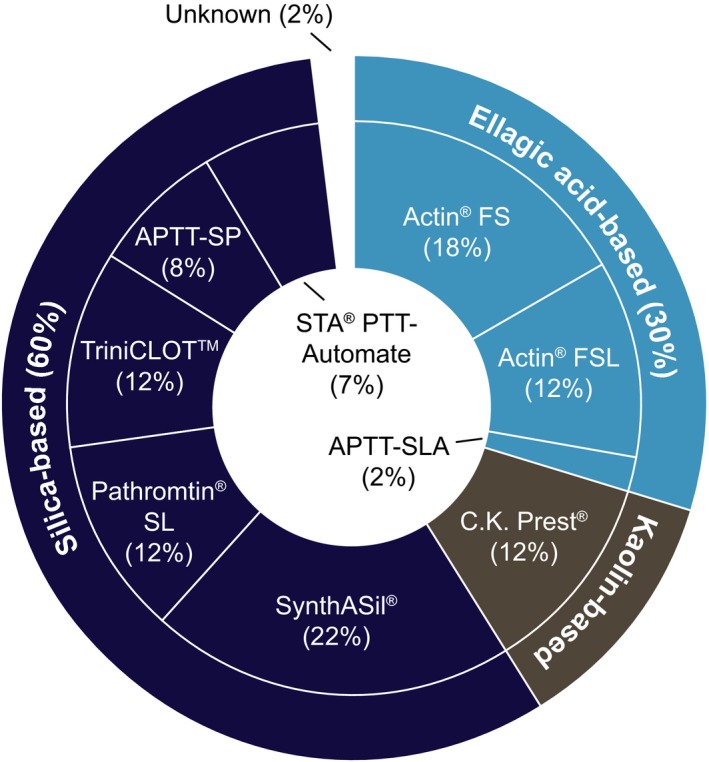
Overview of one‐stage clotting assay aPTT reagents (inner circle) and contact activators (outer circle) tested by participating laboratories (n = 60). Some laboratories reported the use of multiple FVIII:C measurement methods. aPTT, activated partial thromboplastin time; Actin^®^ FS, Actin^®^ FSL, Pathromtin^®^ SL (Siemens Healthcare GmbH); CK Prest^®^, TriniCLOT™, STA^®^ PTT‐Automate (Diagnostica Stago UK Ltd); SynthASil^®^, APTT‐SP (IL); APTT‐SLA (Sysmex)

Laboratories used a total of nine different aPTT reagents in the one‐stage clotting assay. The overall most common silica‐based aPTT reagent was SynthASil^®^, used by 13 laboratories (21.7%). Eighteen laboratories (30.0%) employed aPTT reagents with ellagic acid‐based contact activators, 11 laboratories (18.3%) used Actin^®^ FS, and seven used Actin^®^ FSL (11.7%). Seven laboratories (11.7%) used an aPTT reagent with a kaolin‐based activator, CK Prest^®^ (Figure 1). Survey results for calibration method and sample dilution are summarized in Table 1.

**Table 1:** Analyser, calibration and sample dilution methods routinely used in clinical laboratories

	One‐stage assay	Chromogenic assay
Analyser manufacturer, n (%)		
Siemens	22 (36.7)	11 (30.6)
Instrumentation Laboratory	21 (35.0)	11 (30.6)
Stago	17 (28.3)	9 (25.0)
Calibrator manufacturer, n (%)		
Siemens	26 (43.3)	18 (50.0)
Instrumentation Laboratory	14 (23.3)	3 (8.3)
Stago	12 (20.0)	‐
Precision BioLogic	4 (6.7)	3 (8.3)
NIBSC	‐	4 (11.1)
Homemade	2 (3.3)	2 (5.6)
Hyphen BioMed	‐	3 (8.3)
Sysmex	1 (1.7)	1 (2.8)
Technoclone	‐	2 (5.6)
Unknown	1 (1.7)	‐
Calibration curve, n (%)		
Stored	31 (51.7)	22 (61.1)
Prepared daily	24 (40.0)	11 (30.6)
Diluent for calibration curve, n (%)		
FVIII‐deficient plasma	7 (11.9)	3 (8.6)
Buffer	52 (88.1)	32 (91.4)
Diluent for sample predilution, n (%)		
FVIII‐deficient plasma	3 (5.0)	0 (0.0)
Buffer	55 (91.7)	34 (94.4)
Other	2 (3.3)	2 (5.6)
Number of sample dilutions, n (%)		
Single dilution	23 (38.3)	17 (47.2)
Two dilutions	9 (15.0)	6 (16.7)
Three dilutions	22 (36.7)	11 (30.6)
Four dilutions	1 (1.7)	1 (2.8)
Other	5 (8.3)	1 (2.8)

Abbreviations: FVIII, factor VIII; NIBSC, National Institute of Biological Standards and Control.

### 3.3 One‐stage assay activity measurements

Three of the nine one‐stage assay reagents (ie, APTT‐SP, TriniCLOT™ and STA^®^ PTT‐Automate, all containing silica‐based contact activators) measured N8‐GP activity at approximately 40%‐83% of target concentration (full results are presented in Table 2) and were thus omitted from subsequent statistical analyses for N8‐GP.

**Table 2:** One‐stage aPTT‐based clotting assays that underestimated N8‐GP recovery

aPTT reagent	N8‐GP target concentration (IU/mL)	Mean estimate (% of target)	95% CI (% of target)
APTT‐SP	0.03	56.9	45.1; 68.6
	0.2	59.1	48.5; 69.8
	0.6	58.5	47.9; 69.1
	0.9	56.9	46.3; 67.4
STA^®^ PTT‐Automate	0.03	59.8	40.0; 79.6
	0.2	43.1	22.1; 64.1
	0.6	43.8	22.7; 65.0
	0.9	44.6	23.7; 65.5
TriniCLOT™	0.03	82.9	69.8; 96.0
	0.2	47.7	40.4; 55.1
	0.6	42.6	35.4; 49.9
	0.9	39.7	32.4; 47.0

Abbreviation: CI, confidence interval.

N8‐GP recovery was slightly lower than target concentrations ranging from 101.7% at 0.03 IU/mL concentration to 86.1% at 0.9 IU/mL concentration (Table 3). In contrast, rAHF recovery was higher than target concentrations using one‐stage clotting assays, peaking at 146.1% for 0.03 IU/mL concentration and ranging from 124.2% to 109.1% for 0.2‐0.9 IU/mL concentrations (Table 3). N8‐GP mean recovery remained within acceptable range (±30%) at all concentrations. The overall mean recoveries were 92.5% (95% CI 89%; 96%) of target concentration for N8‐GP and 123% of target concentration (95% CI 120%; 127%) for rAHF.

**Table 3:** One‐stage aPTT‐based clotting assay recovery of N8‐GP and rAHF by target concentration

	Analysis target concentration (IU/mL)	Mean estimate (IU/mL)	95% CI	Mean estimate (% of target)	95% CI (% of target)	Inter‐laboratory CV (%)	Intra‐laboratory CV (%)
N8‐GP	0.03	0.03	0.027; 0.032	101.7	93.7; 109.7	25.5	11.9
	0.2	0.19	0.179; 0.197	95.3	91.5; 99.1	13.1	6.7
	0.6	0.52	0.499; 0.543	87.7	84.4; 91.0	12.3	6.1
	0.9	0.77	0.743; 0.796	86.1	83.3; 88.9	10.9	4.6
rAHF	0.03	0.04	0.040; 0.045	146.1	137.5; 154.7	22.1	12.0
	0.2	0.24	0.234; 0.254	124.2	120.1; 128.4	11.6	11.5
	0.6	0.68	0.660; 0.699	114.2	111.2; 117.2	9.2	8.5
	0.9	0.98	0.956; 0.998	109.1	106.7; 111.4	7.8	6.3
ISTH‐SSC standard lot #4	0.88	0.95	0.92; 0.97	108.6	105.1; 112.0	9.4	6.6

Note

Results from one‐stage clotting assays that used one of the three aPTT reagents (APTT‐SP, TriniCLOT™ and STA^®^ PTT‐Automate) that substantially underestimated N8‐GP recovery were omitted from N8‐GP statistical analysis.

Abbreviations: CI, confidence interval; CV, coefficient of variation; ISTH, International Society on Thrombosis and Haemostasis; SSC, Scientific and Standardization Committee.

**Figure 2:**
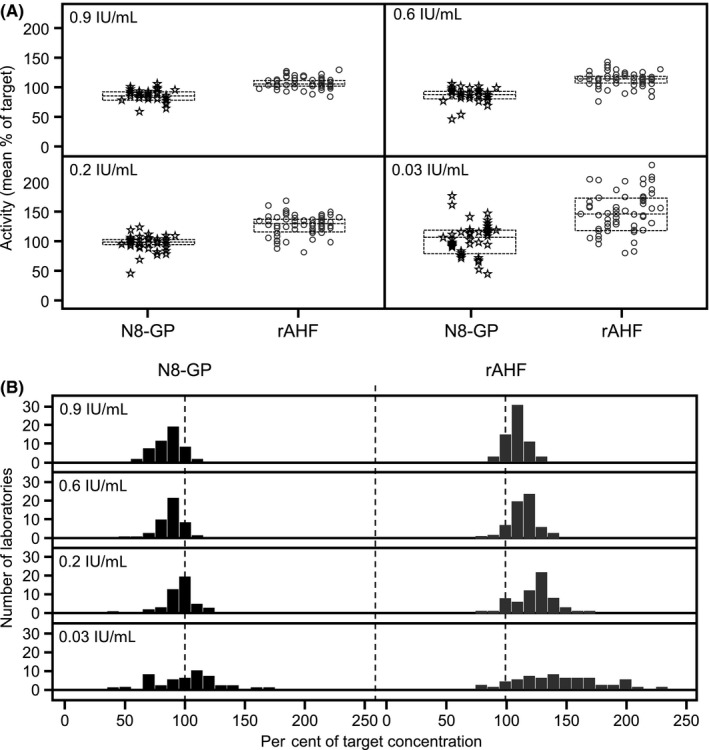
One‐stage aPTT‐based clotting assay mean FVIII:C in plasma samples spiked with 0.03, 0.2, 0.6 and 0.9 IU/mL N8‐GP or rAHF. A, Data points represent mean per cent of target concentration from individual laboratories. Each column of data points represents a different aPTT reagent. Dashed lines represent interquartile range. B, Bars represent the number of laboratories at the mean per cent of target concentration. Results from one‐stage clotting assays that used one of the three aPTT reagents (APTT‐SP, TriniCLOT™ and STA^®^ PTT‐Automate) that underestimated N8‐GP recovery were omitted from N8‐GP statistical analysis, as were values of zero

Both intra‐ and inter‐laboratory variability was similar for N8‐GP and rAHF. The highest inter‐laboratory variability was observed in the ‘very low’ samples (0.03 IU/mL) for both products. Variability decreased with increasing concentration, ranging between 10.9%‐25.5% for N8‐GP and 7.8%‐22.1% for rAHF (Figure 2; Table 3). Furthermore, inter‐laboratory variability in the 0.9 IU/mL samples was similar to SSC lot #4 plasma (0.88 IU/mL) for both products (Table 3).

### 3.4 Chromogenic kits and reagents

**Figure 3:**
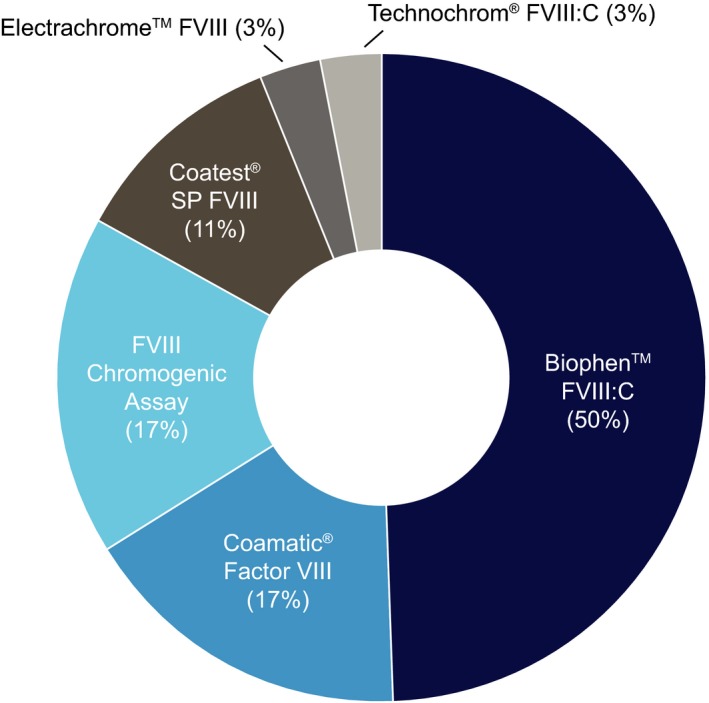
Overview of chromogenic reagents tested by participating laboratories (n = 36). Biophen™ FVIII:C (Hyphen BioMed); Coamatic^®^ Factor VIII, Coatest^®^ SP FVIII (Chromogenix, a brand of IL); FVIII Chromogenic Assay (Siemens); Electrachrome™ FVIII (IL); Technochrom^®^ FVIII:C (Technoclone GmbH)

Overall, 36 laboratories used six different FVIII chromogenic kits, the most commonly used were BIOPHEN™ FVIII:C (50.0%), Coamatic^®^ factor VIII (16.7%) and the Siemens FVIII chromogenic kit (16.7%) (Figure 3). Of the 33 responding laboratories that used chromogenic kits, 22 (66.7%) used stored calibration curves, whereas 11 (33.3%) laboratories calibrated daily (Table 1).

### 3.5 Chromogenic kit activity measurements

**Figure 4:**
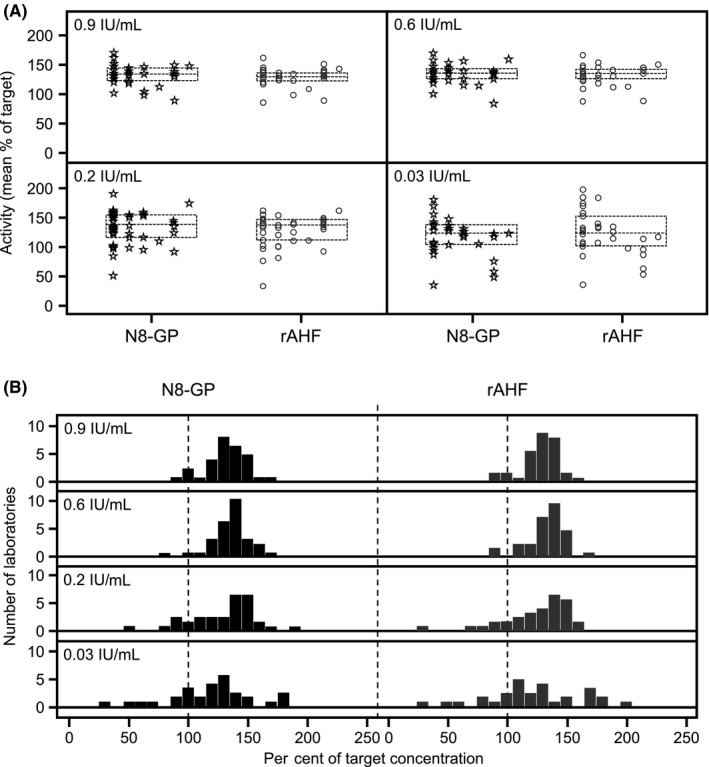
Chromogenic assay mean FVIII:C in plasma samples spiked with 0.03, 0.2, 0.6 and 0.9 IU/mL N8‐GP or rAHF (A) Data points represent mean per cent of target concentration of individual laboratories. Each column of data points represents a different chromogenic kit. Dashed lines represent interquartile range. B, Bars represent the number of laboratories at the mean per cent of target concentration

N8‐GP recovery in the chromogenic assays was similar to rAHF at all concentrations, with both products at concentrations of 0.2 IU/mL and above recovering around 130% of target concentration (Figure 4; Table 4). N8‐GP recovered consistently across all concentrations, and overall, mean recovery was within the upper bound of the acceptable range (129%, 95% CI: 123%; 136%). rAHF mean recovery was similar to that of N8‐GP (127%, 95% CI: 121%; 134%).

**Table 4:** Chromogenic assay recovery of N8‐GP and rAHF by target concentration

Analysis drug	Analysis target concentration (IU/mL)	Mean estimate (IU/mL)	95% CI	Mean estimate (% of target)	95% CI (% of target)	Inter‐laboratory CV (%)	Intra‐laboratory CV (%)
N8‐GP	0.03	0.03	0.029; 0.038	119.1	106.8; 131.4	27.0	18.7
	0.2	0.25	0.23; 0.28	130.5	121.0; 140.0	21.1	7.4
	0.6	0.80	0.77; 0.84	135.0	129.6; 140.4	11.5	4.9
	0.9	1.18	1.13; 1.24	132.7	126.9; 138.4	12.5	4.2
rAHF	0.03	0.03	0.030; 0.039	123.5	109.9; 137.1	29.1	18.5
	0.2	0.24	0.22; 0.27	126.0	116.7; 135.3	21.1	9.5
	0.6	0.79	0.75; 0.82	132.5	127.1; 137.9	11.5	5.6
	0.9	1.14	1.09; 1.19	127.9	122.6; 133.2	11.9	5.4
ISTH‐SSC standard lot #4	0.88	0.94	0.90; 0.98	108.1	104.0; 112.2	10.8	5.8

Abbreviations: CI, confidence interval; CV, coefficient of variation; ISTH, International Society on Thrombosis and Haemostasis; SSC, Scientific and Standardization Committee.

Mean recoveries at 0.03 IU/mL were the closest to target concentration for both products, but, as seen with one‐stage assays, the highest intra‐ and inter‐laboratory variabilities in chromogenic kits were also observed in 0.03 IU/mL samples. Variability decreased with increasing concentration (Figure 4; Table 4). Inter‐laboratory variability was similar between N8‐GP and rAHF, ranging from 11.5% to 27.0% for N8‐GP, and 11.5% to 29.1% for rAHF. Inter‐laboratory variability for SSC lot #4 plasma was similar to high concentration samples of N8‐GP and rAHF (Table 4).

### 3.6 Laboratories that could accurately measure N8‐GP

The three aPTT reagents that underestimated N8‐GP were used in 16 different laboratories, nine of which used both one‐stage and chromogenic assays, of which one of these also used multiple aPTT reagents. Thus, 60 of 67 laboratories (89.6%) routinely used at least one FVIII:C assay that could accurately measure N8‐GP.

## 4 DISCUSSION AND CONCLUSION

Clinical laboratories use a variety of different reagents, methods and assays to monitor FVIII:C in patients treated with FVIII replacement therapy. The recent entry of EHL coagulation factors into clinical practice has introduced the possible benefits of reducing injection frequency and increasing patient trough levels. Treating physicians rely on accurate monitoring of FVIII to ensure that dosing is correct and target trough levels are achieved. Recent studies have reported that some aPTT‐based one‐stage clotting assays inaccurately measure FVIII:C associated with some EHL products.^7^, ^8^ The objective of this global, comparative field study was to evaluate the FVIII:C and assay variability of N8‐GP and rAHF with the methodology and reagents routinely used in clinical laboratories.

In this study, over half of the participating laboratories used chromogenic kits to measure FVIII:C. This is a larger proportion of clinical laboratories than previously reported in other FVIII:C field studies 9, 10, 11 and more in line with results of a survey performed by Kitchen et al^5^ that reported 68% of clinical laboratory scientists used chromogenic kits to measure FVIII:C at least occasionally. Approximately two thirds of laboratories participating in this study were affiliated with the external quality assurance foundation, ECAT, indicating a selection of well‐informed laboratories. This observation may also suggest an increasing global awareness and use of chromogenic kits to measure FVIII:C, especially in light of recent evidence about discrepancies between one‐stage and chromogenic assays in the diagnosis of haemophilia A and B.^18^, ^19^

Overall, most clinical laboratories participating in this study could accurately measure N8‐GP with methods already available in their laboratory, and the recovery for SSC lot #4 obtained using both the chromogenic and one‐stage clotting assays was similar (Tables 3 and 4). Laboratories that deviated most from the assigned value of the SSC lot #4 control sample also provided results that deviated most from the target values for the remaining field study samples (data not shown). Due to regional variability in the availability of many aPTT reagents on the market, local verification of reagents not covered by this survey may be prudent. Although there is currently no consensus about the magnitude of difference from target concentration that is clinically relevant in postinfusion monitoring, many studies define a ±30% difference as acceptable.^10^, ^20^

At 92.5% of target concentration, mean N8‐GP recovery for six aPTT reagents in one‐stage assays was well within the 30% limit. Furthermore, mean N8‐GP recovery was within 30% of target concentration for all spiked samples, nearing 100% of target concentration in the very low concentration sample. However, similar to previous results,^8^, ^21^ this study found that some silica‐based reagents do not recover N8‐GP accurately. Specifically, APTT‐SP, TriniCLOT™ and STA^®^ PTT‐Automate all under‐recovered N8‐GP at about 40%‐83% of target concentration. Recent mechanistic studies indicated that N8‐GP activation by thrombin in specific silica‐based aPTT reagents may proceed slower than compared to unPEGylated FVIII.^22^ This is consistent with studies that found that BAY 94‐9027, a recombinant FVIII with a 60‐kDa PEG moiety, was under‐recovered using select silica‐based aPTT reagents.^7^ Investigators concluded that the PEG moiety interacted with the silica surface, interfering with FVIII activation.^7^

Recoveries using chromogenic kits for both N8‐GP and rAHF in this study were within the upper bound of the acceptable range. This result was obtained with all chromogenic kits tested, except the Technochrome^®^ FVIII:C kit, which was only used by a single laboratory. Slightly higher FVIII:C values are not unexpected and have been previously reported when using chromogenic assays with a normal, pooled plasma calibrator.^12^, ^23^ One possible explanation for the observed difference in recovery of N8‐GP and rAHF is the source of the calibrator used in the potency versus clinical assay. In‐house reference material traceable to the WHO 8th IS FVIII concentrate was used for N8‐GP potency assignment, and this potency assignment was confirmed using various chromogenic kits.^12^ In the clinical laboratories, measurement of FVIII:C is often performed using a chromogenic assay that is calibrated to an in‐house normal, pooled plasma calibrator that is traceable to the WHO 6th IS. In a previous study, we showed that the use of a reference standard traceable to WHO 8th IS FVIII could increase measurement accuracy of chromogenic kits for both N8‐GP and unPEGylated turoctocog alfa (NovoEight^®^). A recent study has shown that chromogenic assays can be validated for use with N8‐GP to give recoveries closer to the expected range when using a normal, pooled plasma calibrator.^24^ Thus, the observed over‐recovery of FVIII:C with chromogenic assays in both EHL and standard rFVIII products deems further and consideration from both clinical laboratory scientists and assay manufacturers.

Overall, most participating clinical laboratories could accurately measure N8‐GP and rAHF using their in‐house available one‐stage clotting or chromogenic FVIII:C assays, without the need of a product‐specific standard. Three silica‐based aPTT reagents substantially underestimated N8‐GP recovery and should not be used to monitor N8‐GP activity.

## DISCLOSURES

M. Ezban, W.H.O. Clausen, M. Hansen, and R. Lützhøft are employees or former employees and shareholders of Novo Nordisk A/S. S. Tiefenbacher is an employee and shareholder of the Laboratory Corporation of America Holdings. He has received honoraria or consultation fees from Novo Nordisk A/S, Siemens Healthcare and Shire.
