# Supplementary material for: A field study evaluating the activity of N8‐GP in spiked plasma samples at clinical haemostasis laboratories
Source: Haemophilia. 2019 Jul 11;25(5):893–901. doi: 10.1111/hae.13813 (PMC6852407; doi:10.1111/hae.13813)
Supplement: Supplementary file 1 [file HAE-25-893-s001.docx]

Supplemental Table 1. Geographical distribution of laboratories by assay type

|  |  | Number of laboratories using assay type | | |
| --- | --- | --- | --- | --- |
| Country | Total assays (%) | One-stage* | Chromogenic | Both |
| **All** | **67 (100)** | **60** | **36** | **29** |
| France | 11 (16.4) | 8 | 6 | 3 |
| USA | 8 (11.9) | 8 | 4 | 4 |
| UK | 7 (10.4) | 6 | 7 | 6 |
| Netherlands | 5 (7.5) | 5 | 2 | 2 |
| Australia | 4 (6.0) | 4 | 3 | 3 |
| Canada | 4 (6.0) | 4 | 2 | 2 |
| Japan | 4( 6.0) | 4 | 1 | 1 |
| Switzerland | 3( 4.5) | 3 | 1 | 1 |
| Austria | 2 (3.0) | 2 | - | - |
| Belgium | 2 (3.0) | 2 | 1 | 1 |
| Spain | 2 (3.0) | - | 2 | - |
| Sweden | 2 (3.0) | 1 | 2 | 1 |
| Brazil | 1 (1.5) | 1 | 1 | 1 |
| Denmark | 1 (1.5) | 1 | 1 | 1 |
| Finland | 1 (1.5) | 1 | 1 | 1 |
| Germany | 1 (1.5) | 1 | 1 | 1 |
| Greece | 1 (1.5) | 1 | - | - |
| India | 1 (1.5) | 1 | 1 | 1 |
| New Zealand | 1 (1.5) | 1 | - | - |
| Poland | 1 (1.5) | 1 | - | - |
| Portugal | 1 (1.5) | 1 | - | - |
| South Africa | 1 (1.5) | 1 | - | - |
| Taiwan | 1 (1.5) | 1 | - | - |
| Thailand | 1 (1.5) | 1 | - | - |
| Turkey | 1 (1.5) | 1 | - | - |
| *One laboratory in the UK used three aPPT reagents; one laboratory in France used two aPTT reagents. | | | | |
